# Low-Power Analog Processing for Sensing Applications: Low-Frequency Harmonic Signal Classification

**DOI:** 10.3390/s130809604

**Published:** 2013-07-25

**Authors:** Daniel J. White, Peter E. William, Michael W. Hoffman, Sina Balkir

**Affiliations:** 1 Department of Electrical Engineering, University of Nebraska–Lincoln, Lincoln, NE 68588 0511, USA; E-Mails: mhoffman1@unl.edu (M.W.H.); sbalkir@unl.edu (S.B.); 2 Phillips Healthcare, 1 Echo Drive, Reedsville, PA 17084, USA; E-Mail: peter.william@philips.com

**Keywords:** Analog Harmonic Transform, classification, mixed-signal, low-power

## Abstract

A low-power analog sensor front-end is described that reduces the energy required to extract environmental sensing spectral features without using Fast Fouriér Transform (FFT) or wavelet transforms. An Analog Harmonic Transform (AHT) allows selection of only the features needed by the back-end, in contrast to the FFT, where all coefficients must be calculated simultaneously. We also show that the FFT coefficients can be easily calculated from the AHT results by a simple back-substitution. The scheme is tailored for low-power, parallel analog implementation in an integrated circuit (IC). Two different applications are tested with an ideal front-end model and compared to existing studies with the same data sets. Results from the military vehicle classification and identification of machine-bearing fault applications shows that the front-end suits a wide range of harmonic signal sources. Analog-related errors are modeled to evaluate the feasibility of and to set design parameters for an IC implementation to maintain good system-level performance. Design of a preliminary transistor-level integrator circuit in a 0.13 *μ*m complementary metal-oxide-silicon (CMOS) integrated circuit process showed the ability to use online self-calibration to reduce fabrication errors to a sufficiently low level. Estimated power dissipation is about three orders of magnitude less than similar vehicle classification systems that use commercially available FFT spectral extraction.

## 1. Introduction

Sensor systems typically operate by transducing some physical quantity (e.g., pressure, velocity, flux) into the electrical domain and applying signal conditioning. They then compute “features” of the sensed signal relevant to its cause and make decisions or perform actions as a result of the extracted information. Because digital computers are best suited for back-end information processing and decision-making tasks, there must be an analog-to-digital conversion (ADC) as part of the system-level operation. Where in the processing chain the dofor digital operations. The magnitude main conversion happens can affect implementation characteristics such as hardware complexity, energy consumption and service lifetime. Our long-term goal is a very low-power, single-chip, multi-modal environmental sensor that contains a micro-processor and flexible analog signal processing blocks.

The majority of signal detection and classification schemes first transform the acquired signal into a representation that can reveal significant characteristics in a relatively condensed feature vector. Fouriér and wavelet transforms have been proven to generate suitable features for many signal detection and classification tasks, including speech recognition [1], vehicle detection and classification [2–5], and bearing fault detection [6–9]. Several low power monitoring schemes use statistical parameters as features, such as signal mean, standard deviation and peak. These parameters are obtained directly from the time domain signal to generate signal features for identification [10], but they are limited in the amount of classification information they contain compared with spectral features. Discrete Fouriér and wavelet features require sampling the signal at relatively high rates prior to processing.

Compressive Sensing (CS) has been proposed as an efficient alternative to high-rate Nyquist sampling [11]. It relies on the signal being sparse in some domain to achieve its gains, extracting signal characteristics using projection over random basis functions. Signal features obtained using CS are randomly distributed across the projected data and are not directly usable for discrimination. The data must be transformed again into a suitable domain using complex algorithms that are not suitable for energy-constrained sensor modules.

Hardware-oriented architectures and designs utilizing CS concepts have recently been proposed in [12–16]. These techniques are targeted at sampling high-frequency communication signals over large bandwidths with the goal of reducing the ADC rate. They trade off ADC rate-reduction for a large increase in back-end computation required to reconstruct the sensed spectrum from the acquired random projections.

While extracting spectral information with the Fast Fouriér Transform (FFT) is a common method, its disadvantages are apparent within an energy-or power-constrained sensor node. As an example, energy usage data for a military vehicle classification application was measured in [17]. It extracted features from the signal's spectrum with a 512-sample FFT and used a Support Vector Machine classifier. Implemented on a Mica2 sensor node, the FFT computation consumed 5.1mJ of energy, while the feature selection and classification only consumed 0.1 mJ, an order of magnitude less. Thus, reduction of the energy required to extract spectral features has the potential to make a large impact on a sensor node's energy budget.

**Figure 1.**
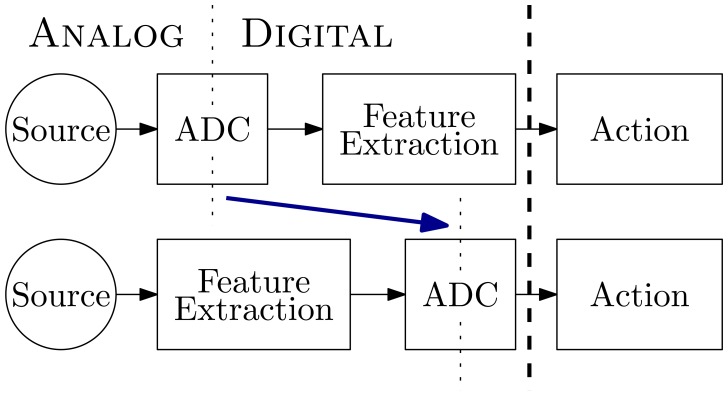
Performing the feature extraction operation in the analog domain moves the system's analog-to-digital conversion (ADC) later.

Directly extracting spectral information in the analog domain bypasses the FFT computation and allows selectively converting features as needed. Figure 1 illustrates exchanging the ADC and feature extraction steps to perform the domain conversion on the spectral features instead of the raw signal. Moving the analog-digital boundary can provide a good match between the computations performed and the underlying device characteristics, yielding efficient computation [18].

In this paper, an Analog Harmonic Transform (AHT) is developed, which extracts feature vectors directly from acquired analog signals. These features may then be used for signal classification or other back-end processing, either directly or transformed into equivalent Fouriér series coefficients by a simple back-substitution. This transform replaces the typical ADC/FFT with a multi-channel analog projection to extract a signal's spectral features.

The outline of the paper is as follows. In Section 2, we introduce the AHT and its relationship to the Fouriér series for performing the feature extraction function within an analog front-end, with emphasis on power consumption and overall system performance. Two case studies are presented in Section 3 to validate the approach within a classification system using ideal calculations. Section 4 describes a hardware realization of the AHT, modeling analog-related errors to determine the feasibility of, and set design specifications for, a hardware design. The paper concludes with Section 5.

## 2. Analog Harmonic Transform

**Figure 2.**
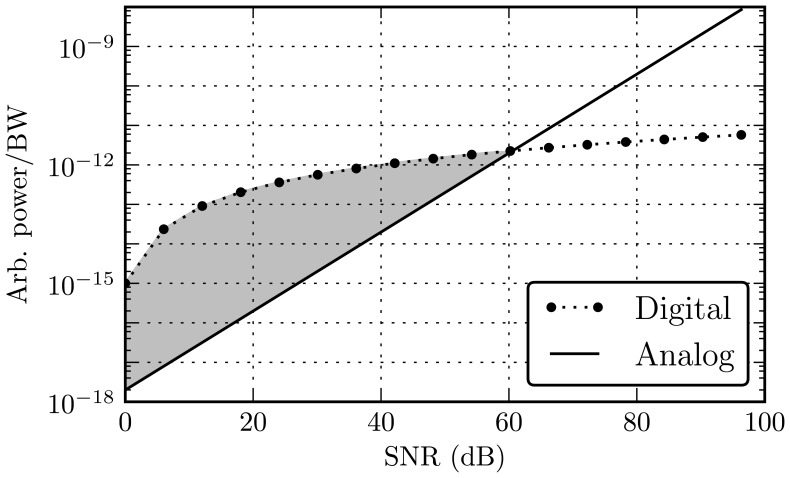
Analog and digital power requirements for signal processing as a function of signal-to-noise ratio (SNR). Power is in arbitrary units and normalized to signal bandwidth.

Comparisons of the power and area required to implement signal-processing operations at a given precision between analog or digital integrated circuitry have been described by [18,19] and others. Figure 2 plots the shape of power requirements for digital and analog computation *versus* precision, given as signal-to-noise ratio (SNR), is varied. Both power and area scale linearly with SNR for analog and as log_2_(SNR) for digital operations. The magnitude and crossover point depends on factors, such as task, technology and the skill level of the designers [18].

Clearly, from Figure 2, applications requiring high precision computation are best served by digital systems. However, systems that can tolerate lower SNRs can utilize an analog implementation's fundamental energy advantage, especially with SNRs below about 40 dB, indicated by the shaded region. The challenge for energy-efficient signal processing systems is then to find algorithms and architectures that maintain good system-level performance with low precision or noisy computations. The AHT focuses on the feature extraction phase of harmonic signal classification tasks to take advantage of analog techniques, as suggested by the energy usage data from [17].

### 2.1. Harmonic Signals

Sensed harmonic signals originating from rotating machinery may be modeled as a sum of two components: a deterministic harmonic signal model approximating the revolving parts and a non-deterministic component approximating all other components. Selective features extracted from these signals are sufficient for signal/source discrimination, as shown in [3-5]. A harmonic signal can be described as:

(1) $$x(t)=\overset{M}{\underset{k=1}{\text{∑}}}\alpha_{k}\cos(2\pi kf_{1}t+\phi_{k})+n(t)$$

where *α_k_* and *ø_k_* are the amplitude and phase of the *k*th deterministic harmonic component, respectively, *f*_1_ is the fundamental frequency (FF), *M* is the largest harmonic number and *n*(*t*) is the non-deterministic signal component. The signal's harmonic part is therefore completely defined by 2*M*+1 parameters. If the FF and number of harmonics are known, the optimum solution in additive white noise for estimating the amplitude and phase set is the least squares (LS) solution, *i.e.*, the signal's Fouriér series (FS) coefficients. An alternate solution for estimating the harmonic parameters is to locate spectral peaks that maintain a line series, presented in [20,21] as Harmonic Line Association (HLA). HLA, however, requires narrow frequency resolution (long FFT) and a complex approach for the selection of harmonically related peaks. In contrast, the time domain harmonics' amplitudes (TDHA) method extracts harmonic signal information with lower complexity than the FFT [5], but still operates in the digital domain and requires multiplication. We now describe a new transform for calculating these harmonic parameters well-suited for efficient analog-domain implementation.

### 2.2. Analog Basis Projection

To estimate the *k*th harmonic's amplitude, *α_k_*, and phase *ø_k_*, the input signal is first low-pass filtered to *Mf*_1_ and, then, projected onto a pair of quadrature basis functions with frequency *kf*_1_ and integrated over T= 1/*f*_1_ as:

(2) $$\begin{array}{l} y_{Ik}=\int_{0}^{T}x(t)\psi_{Ik}(t)dt\\ y_{Qk}=\int_{0}^{T}x(t)\psi_{Qk}(t)dt \end{array}$$

where *k*∈ {1, 2, …, *M*}, with basis functions given as:

(3) $$\begin{array}{l} \psi_{Ik}(t)=\mathrm{sgn}(\cos(2\pi kf_{1}t))\\ \psi_{Qk}(t)=\mathrm{sgn}(\sin(2\pi kf_{1}t)) \end{array}$$

**Figure 3.**
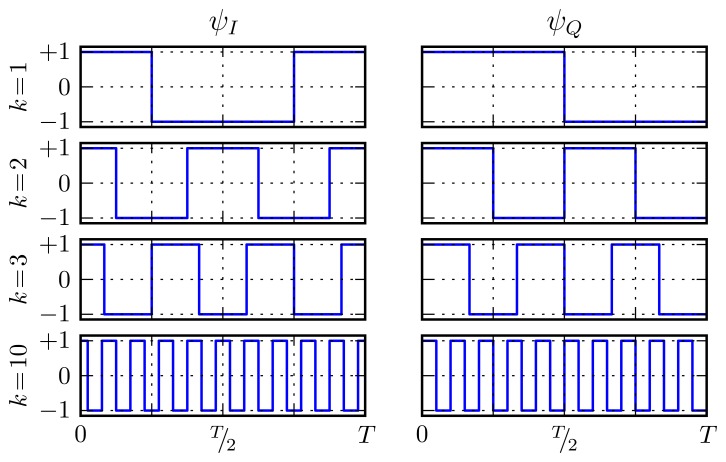
Quadrature basis function waveforms for *k*= 1, 2, 3, 10.

The AHT scheme takes the Fouriér series' sinusoidal basis functions and uses only their signs, as shown in Equation (3). This change greatly simplifies analog implementation of the projection implementation, as explained in Section 4. Figure 3 plots the basis pairs for harmonic numbers, 1, 2, 3, and 10, with $f_{1}=\frac{1}{T}$.

### 2.3. Feature Extraction

The harmonic part of the signal in Equation (1) can be expressed as in-phase and quadrature components by:

(4) $$x(t)=\overset{M}{\underset{k=1}{\text{∑}}}\alpha_{k}\cos(\phi_{k})\cos(2\pi \text{kft})-\overset{M}{\underset{k=1}{\text{∑}}}\alpha_{k}\sin(\phi_{k})\sin(2\pi \text{kft})$$

Substituting Equations (3) and (4) into Equation (2) and evaluating the integration gives:

(5) $$\begin{array}{l} y_{Ik}=\frac{T}{2\pi }\overset{M}{\underset{p=1}{\text{∑}}}\frac{\alpha_{p}\cos(\phi_{p})}{p}\overset{2k}{\underset{r=1}{\text{∑}}}{\left(-1\right)}^{r-1}\sin(\frac{(2r-1)\pi p}{2k})\\ y_{Qk}=\frac{-T}{2\pi }\overset{M}{\underset{p=1}{\text{∑}}}\frac{\alpha_{p}\sin(\phi_{p})}{p}\overset{2k}{\underset{r=1}{\text{∑}}}{\left(-1\right)}^{r-1}\cos(\frac{2r\pi k}{2p}) \end{array}$$

The result of each in-phase and quadrature projection represents the sum of scaled in-phase and quadrature harmonics' amplitudes, respectively. To better illustrate the relationship between the harmonic parameters (*α_k_*, *ø_k_*) and the basis projections (*y_Ik_*, *y_Qk_*), the parameter sets can be represented in the column vector format as:

(6) $$\begin{array}{ll} y_{I}=\{y_{Ik}\} & y_{Q}=\{y_{Qk}\}\\ {\text{a}}_{I}=\{a_{Ip}=\alpha_{p}\cos(\phi_{p})\} & a_{Q}=\{a_{Qk}=\alpha_{p}\sin(\phi_{p})\} \end{array}$$

The relation may then be written as:

(7) $$\begin{array}{c} y_{I}=\frac{T}{2\pi }U_{I}a_{I}\\ y_{Q}=-\frac{T}{2\pi }U_{Q}a_{Q} \end{array}$$

with:

(8) $$U_{I}=[\begin{array}{ccccccccccc} 1 & 0 & -\frac{1}{3} & 0 & \frac{1}{5} & 0 & -\frac{1}{7} & 0 & \frac{1}{9} & 0 & \ldots \\ 0 & 1 & 0 & 0 & 0 & -\frac{1}{3} & 0 & 0 & 0 & \frac{1}{5} & \ldots \\ 0 & 0 & 1 & 0 & 0 & 0 & 0 & 0 & -\frac{1}{3} & 0 & \ldots \\ \vdots & & & & & \ddots & & & & & \vdots \\ 0 & 0 & 0 & \ldots & \ldots & \ldots & . & 0 & 0 & 0 & 1 \end{array}]$$

(9) $$U_{Q}=[\begin{array}{ccccccccccc} 1 & 0 & \frac{1}{3} & 0 & \frac{1}{5} & 0 & \frac{1}{7} & 0 & \frac{1}{9} & 0 & \ldots \\ 0 & 1 & 0 & 0 & 0 & \frac{1}{3} & 0 & 0 & 0 & \frac{1}{5} & \ldots \\ 0 & 0 & 1 & 0 & 0 & 0 & 0 & 0 & \frac{1}{3} & 0 & \ldots \\ \vdots & & & & & \ddots & & & & & \vdots \\ 0 & 0 & 0 & \ldots & \ldots & \ldots & . & 0 & 0 & 0 & 1 \end{array}]$$

The in-phase and quadrature amplitude vectors may then be calculated by:

(10) $$\begin{array}{ll} a_{I}={U_{I}}^{-1}y_{I} & \;a_{Q}={U_{Q}}^{-1}y_{Q} \end{array}$$

Individual harmonic magnitude and phase estimates may be calculated from the rectangular parameters via:

(11) $$\begin{array}{ll} \alpha_{k}=\sqrt{a_{Ik}^{2}+a_{Qk}^{2}} & \;\phi_{k}=\tan^{-1}\left(\frac{a_{Qk}}{a_{Ik}}\right) \end{array}$$

### 2.4. Computational Considerations

**Figure 4.**
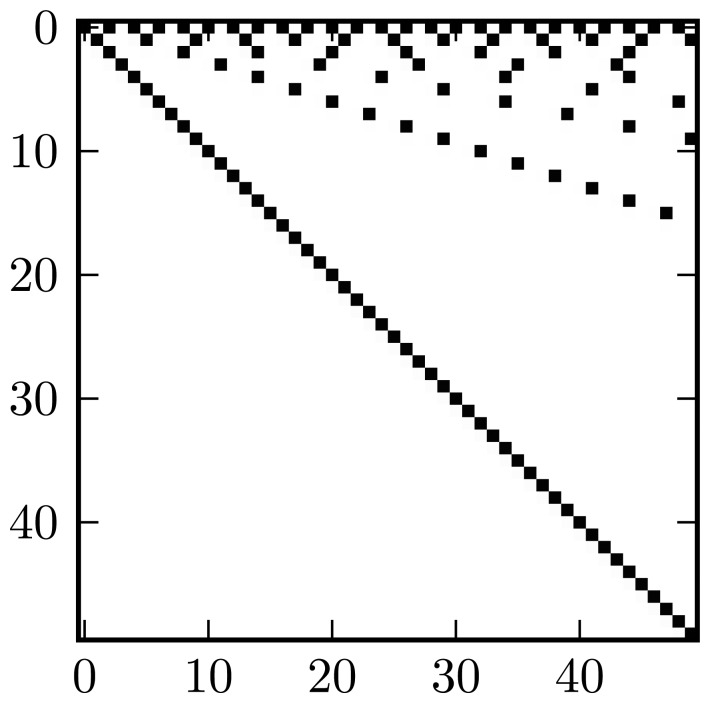
Structure of matrices *U_I_*_,_*_Q_*; non-zero entries are black.

Matrices ***U****_I_* and ***U****_Q_* are sparse, upper-triangular and unipotent. These properties ensure their inverses are well-conditioned and independent of any signal characteristics for a given *M*. From the upper-triangular property, *a_Ik_* and *a_Qk_* may be calculated by a simplified back-substitution, while the sparsity greatly reduces the number of computations actually required. Figure 4 shows the non-zero matrix entries.

To illustrate the digital computation savings, Table 1 lists the number of real-valued multiplications and additions to recover the Fouriér series coefficients from the AHT projected values and compares with the equivalent FFT operation to yield the same coefficient set. The number of operations required for the FFT may be reduced by exploiting the fact that the input data is purely real. The efficient Real Split-Radix FFT (RSR-FFT) algorithm discussed in [22] is therefore used for comparison purposes. Operation counts for the FFTs assume three multiplications and three additions per complex multiply by using Gauss' algorithm and observe that two of the additions may be pre-computed, due to the constant twiddle factors [22]. The AHT back-substitution uses real values only.

Unlike the FFT, which produces all coefficients in the signal's bandwidth, it is not necessary to calculate all the AHT coefficients if the back-end application will not use them. For example, if the lower 5% of the coefficients (three harmonics for *M* = 64) do not increase the system-level performance, they need not be calculated. Due to the *U_I_*_,_ *_Q_* matrix's structure, this would reduce the number of multiplication and addition operations by 30% and 58%, respectively, to calculate the top 95% of the coefficients. Advanced energy-aware detection and classification algorithms may selectively disable the unneeded harmonic projection channels for a further reduction in energy usage. Such fine-grained, adaptive energy management is not possible when generating frequency coefficients using FFT only.

Finally, it has been shown in [23] that calculating a pseudo-amplitude as $\sqrt{y_{Ik}^{2}+y_{Qk}^{2}}$ in place of the FS amplitudes results in minimal system-level classification performance degradation. This means that the AHT coefficient to FS coefficient calculation may be skipped altogether, with its attendant energy savings.

## 3. Ideal System Evaluation

It is first necessary to determine if this transform provides useful information for a back-end classifier before considering its analog implementation. Two case studies are presented to validate the use of the AHT for harmonic signal classification applications. The first is classification of vehicle types from acoustic recordings, while the second is the identification of machine bearing faults from acceleration data. System classification performance for both applications will be shown to be comparable to existing studies using considerably more complex front-end processing techniques on the same data sets.

### 3.1. Case Study I: Classification of Military Vehicles

Monitoring large regions for military vehicle activity for peacekeeping purposes is an application well-suited for wireless sensor modules. The acoustic emissions of such ground vehicles contain a wealth of information for purposes such as classification [24]. The main sources of acoustic emissions are from the engine and propulsion mechanism and can be approximated using a harmonic signal model [25,26].

**Figure 5.**
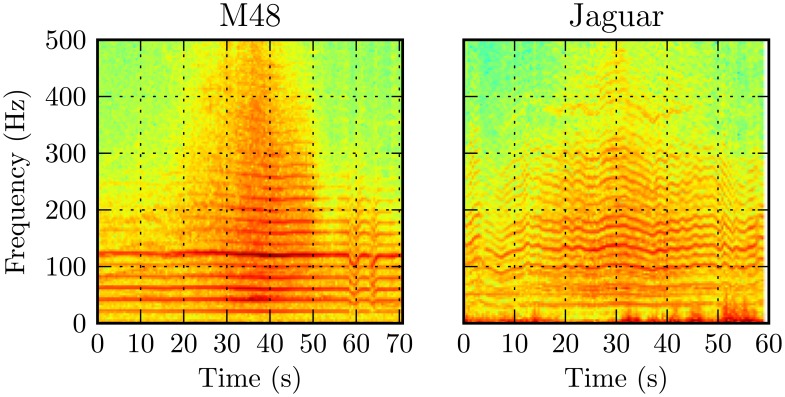
Time-frequency acoustic response of two ground military vehicles.

For ground vehicles, the engine-related FF of the acoustic signal typically lies within the range, 8–20 Hz [21,25]. The time-frequency responses of sample runs of the acquired acoustic signals from two tracked military vehicles passing by a sensor node are shown in Figure 5. The harmonic structure and the time-varying nature of the signals are apparent.

The acoustic data of nine different vehicles covering all combinations of wheeled/tracked and heavy/light-weight types (Leopard 1, Leopard 2, Wiesel, Jaguar, M48, Fuchs, Hermelin, Unimog, and Mercedes-Benz 1017) were recorded by the Bochum Verification Project (BVP) during verification experiments in 2000 [3,27]. The researchers equipped each of two stations with acoustic and seismic sensors to record signatures of the vehicles driving along four different lanes (paved and unpaved). Only the acoustic data is considered in this case study, since the harmonic structure is more pronounced. Each run represents one vehicle passing by two stations placed 101.4 meters apart on opposite sides of the lanes. More than 365 runs were recorded at variable speeds, from different directions and on different surfaces. Each recording was started manually when the vehicle entered within 200 m of the sensor stations.

To determine the presence of a vehicle, an adaptive Constant False Alarm Rate (CFAR) detector [5] was used to output a decision every 0.5s, based on the average energy level of the acoustic signal. As an energy-based detector, it also detected events with no clear harmonic signature. Note that these events are included in the classification rate in the results presented. At normal speeds, medium and lightweight vehicles were detected within 50 m of the sensor station, while heavyweight tracked vehicles were detected beyond 100m. Due to this variation, the total number of detection events per vehicle ranged between 1,200 and 5,500.

For classification, a three-layer feed-forward neural network (FNN) was utilized with sigmoid neuron transfer functions. Harmonic amplitudes from each window (*a_k_* or *α_k_)* were used as the feature vectors and fed as the FNN input layer. Forty hidden neurons made up the middle layer, while the output layer consisted of nine neurons representing each of the vehicles. The network was trained using the resilient backpropagation algorithm (Rprop) [28]. For all results, $\frac{1}{3}$ of the total number of detected events were randomly selected for network training with the remaining $\frac{2}{3}$ used for testing.

Harmonic amplitudes were calculated using the AHT of Section 2.2 with three harmonic models, each with an assumed $\hat{FF}=5\text{Hz}$ (less than half the expected range of 8–20 Hz) and *M* = {25, 50,100}. This approximated the deterministic signature in the bands, 5–125 Hz, 5–250 Hz and 5–500 Hz, respectively. Single-event detection, false alarm and classification rates are shown in Table 2. Military vehicle acoustic signature single-event classification rates of ≥ 80% are considered excellent [29].

From Table 2, we conclude that the transform was capable of extracting distinctive features sufficient for acceptable vehicle discrimination. Note that this was without estimation of the fundamental frequency or exact number of harmonics. For constant spectral resolution (5 Hz), the capability to discriminate among military vehicles using harmonic amplitudes increased with bandwidth (increasing *M*) up to 250 Hz. Further increase in bandwidth beyond 250 Hz gave little improvement. This matches with the sample spectrograms in Figure 5, which show little signal energy above 250 Hz, except when the vehicle is passing very near the station. Separate studies reducing the spectral resolution below 5 Hz for the same bandwidths did not yield significantly better classification rates considering the increase in feature vector length [23].

Previously published classification results from this data set include the original research [3] and more recent work [5]. The first study extracted spectral information with the FFT and employed learning vector quantization (LVQ) for classification. For 5Hz resolution, a 88.02% average correct single-event classification rate was achieved. Both estimated and fixed fundamental frequencies were used with the time-domain harmonic amplitude (TDHA) spectral extraction of [5]; the classification rate with a fixed $\hat{FF}$ of 5 Hz was 85.20%, while using an estimated FF close to 5 Hz raised the rate to 90.38%. Feature vectors obtained from the first 50 coefficients of a 5 kHz-sampled, 1,024-point FFT (4.88 Hz resolution) were also evaluated in [5] and gave an 88.02% classification rate. These results are comparable to the 88.14% single-event classification rate achieved here, but with substantial reduction in computation complexity, especially compared with FFT. Extensive cross-validation studies were done between randomly-selected sets of events for training and testing similar to those done in [5]. The processing approach described in this work presents comparable performance with the promise of much lower power requirements.

### 3.2. Case Study II: Identification of Bearing Faults in Rotating Machinery

Induction motor failures may be classified as bearing, stator, broken rotor bar, end ring or eccentricity-related faults [9]. These faults may lead to increased vibration and noise levels and can be detected by monitoring machine vibrations, acoustic emissions or motor current signals.

Unlike the other fault classes, which have signatures directly related to shaft speed, bearing-related faults are difficult to represent with a single harmonic model. The natural mechanical resonance frequencies of the machine are modulated by the defect frequency, resulting in spectral components that are not harmonics of either the defect frequency or the machine's natural resonance frequencies [9]. The defect frequencies generated from specific faults depend on their location within the bearing structure (inner or outer race, ball, cage) and the bearing assembly's geometry. Amplitudes of the defect frequencies have been shown to be an indication of the severity in [7].

**Figure 6.**
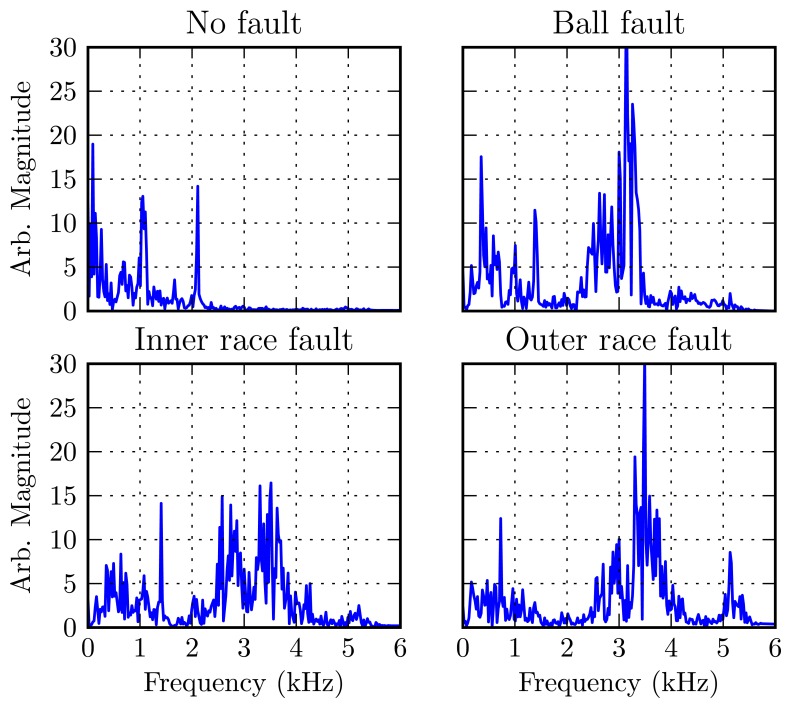
Vibration spectra of an electric motor with various drive-end bearing faults at 1,772 RPM and 2HP load.

The data set from [30] is a collection of accelerometer data from the introduction of single-point faults to test bearings mounted in a three-horsepower induction motor. Bearings were separately prepared with 7, 14, and 21 mil diameter faults on a ball, inner race or outer race. The signal from an accelerometer mounted on the motor housing at the drive end was recorded at motor loads of 0–3 HP. Figure 6 shows representative vibration spectra of the signals for normal operation and with the different bearing fault types.

Under normal operation, most energy is concentrated below about 2 kHz, while the presence of faults moves this energy into the 2–4 kHz band. A single harmonic model with $\hat{FF}=100\;\text{kHz}$ was chosen to give sufficient resolution to extract the spectral envelope without attempting to identify and match individual intermodulation components. A similar neural network and training procedure to the one used in Case Study I in Section 3.1 was used for classification of the bearing fault.

Table 3 shows the classification results for three values of *M* = {10, 20, 40}, corresponding to upper frequencies of 1, 2 and 4 kHz, respectively. It is clear that the lower 10 harmonic amplitudes are sufficient for discriminating a healthy bearing, but not for identifying the type of defect. To identify the defect type, at least 20 harmonics are required to reliably approximate motor vibrations. A harmonic model with more than 40 harmonics has little advantage, since there was little vibration energy above 4 kHz. Previously, published classification rates using this data set were in the range of 82.8–100%, as summarized in [31].

## 4. Feasibility of Hardware Implementation

**Figure 7.**
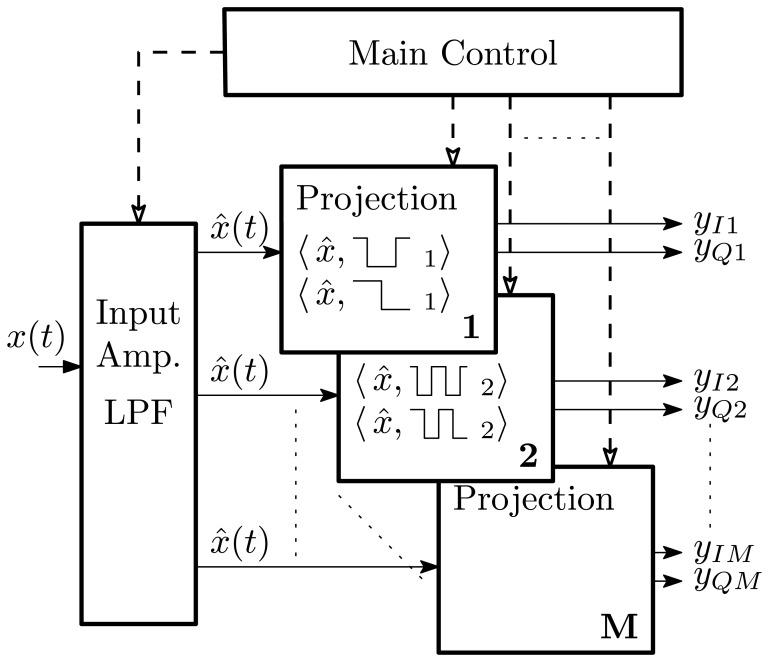
Analog projection system block diagram.

The transform scheme presented in Section 2 has features well-suited to analog-domain implementation. The results of using this approach in Section 3 show that it is competitive in terms of performance with state-of-the-art techniques, while presenting the promise of very low-power analog implementations. This section explores an example system, which exploits these analog-friendly features and extracts relevant hardware specifications that would be required to maintain good system-level performance. Figure 7 shows a top-level block diagram of such a system. The input amplifier low-pass filters the signal to the maximum expected harmonic frequency and distributes the resulting signal to the projection blocks. Each harmonic projection shares the common input and global timing signals and receives individual configuration information from the main control. The circuitry for each projection block can then be identical, allowing a highly regular circuit implementation to minimize inter-channel differences. Following the projection interval, each of the 2*M* values may be read by an ADC, as required by the system, typically serially through an analog multiplexer.

### 4.1. Transform Features and Architecture

**Figure 8.**
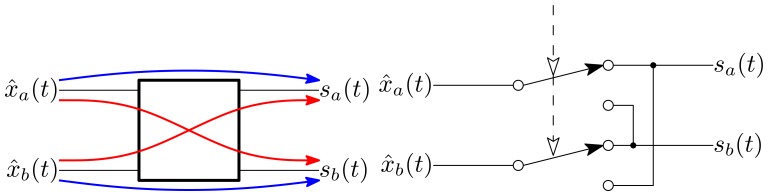
Differential ±1 multiplier.

Multiplication of the input signal by the basis function values of ±1 may be viewed as a conditional signal pass-through or inversion. Using a differential signal path, this inversion is simply a re-labeling of the signal branches, as illustrated in Figure 8. This reduces the signal-basis multiplication operation to a double-pole double-throw switch, which is readily implemented with analog switches. Without this simplification, the necessary continuous-valued four-quadrant analog multiplier would dominate the noise and distortion performance of the signal path. In addition, the real-time basis function generation circuitry does not need to create synchronized sets of quadrature sinusoids of sufficient purity. The required switch timing signals may be readily generated by several analog or digital techniques, such as multiplying phase-locked loops or numerically-controlled oscillators (NCO).

**Figure 9.**
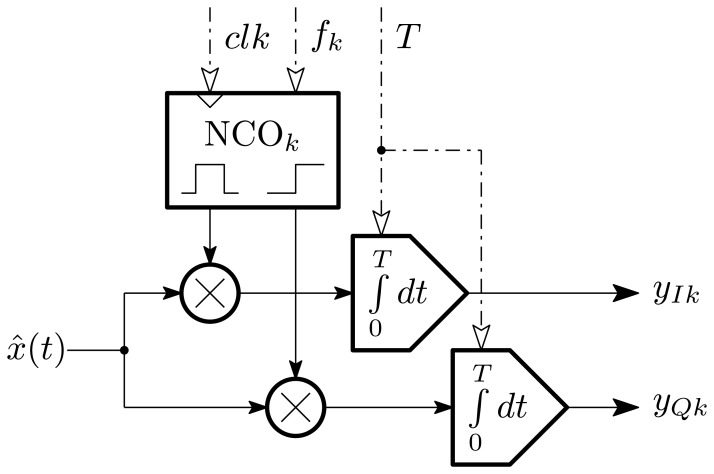
Harmonic projection channel block diagram.

Figure 9 shows the contents of each harmonic projection block. A digital NCO is shown generating the basis functions under control of a system clock and frequency control word. The modulated signal is then integrated by a number of techniques, which could be as simple as a single-pole filter of appropriate time constant. Depending on the integrator implementation, the time constant can be made to span orders of magnitude, supporting applications with wide-ranging fundamental frequencies, such as vehicle classification (8–20 Hz) and bearing fault detection (100–1,000 Hz) with the same circuitry.

Other projection systems use a similar hardware topology, but employ Compressive Sensing concepts for basis function generation [12,13,16,32]. However, the basis function generators in [12] must be operated at greater than twice the maximum signal frequency to achieve the required randomness for CS-based reconstruction. The chipping rate can be reduced to sub-Nyquist [15] under certain signal-dependent assumptions, but is still tied to the signal's maximum frequency. Here, the basis functions are always less than or equal to the maximum signal frequency and equal to the desired harmonic frequency, which is, in general, substantially lower than the maximum frequency. Furthermore, the frequency (FS coefficients) and time-domain waveforms are readily calculated in this scheme from the output vector without requiring the complex reconstruction operation (e.g., basis pursuit) inherent in the CS paradigm.

Furthermore, featured in this topology are the low bandwidth requirements placed on the active circuitry; only the input buffer amplifier must operate over the entire signal bandwidth. The integrators in the projection blocks only need response on the order of the integration time window, *T*. Power dissipation in many integrators is inversely proportional to their time constant, leading to inherently low-power operation.

### 4.2. Hardware Error Sources

Errors introduced into the computation of Equation (2) by hardware may result in degraded system-level performance. The effects of non-ideal computation must be accounted for in order to both determine the system's feasibility and to set the required hardware design specifications, which maintain acceptable system-level performance. These errors may be combined into five classes: timing, distortion, gain, offset and random noise.

- **Timing Errors:** These errors come from basis function generation and reset/readout delays in the integrators. Jitter in the basis function waveforms broadens the spectral sensitivity of the channel. For an NCO implementation, employing a sufficient number of phase accumulator bits and reducing the ratio of highest frequency harmonic to digital clock rate, *f_M_*/*f_clk_*, can render these errors insignificant.
- **Distortion Errors:** Waveform distortion from input amplifier and integrator nonlinearity generates additional signal-related frequency content in addition to that of the original input signal. The net effect of this is additional terms in the matrices, ***U_I_*_,_*_Q_***, in Equation (7) below the diagonal entries, invalidating its upper-triangular property and compression of the diagonal entries at large input amplitudes.
- **Gain Errors:** Gain errors arise from unequal amplifier gains from the input to the individual projection blocks and integrator time constant variations; the latter may vary by as much as a factor of three with poor design and physical layout. The cumulative effect is a random scale factor for each harmonic amplitude. Due to their nature, these errors may be considered fixed for a given IC sample and operating conditions and can be mitigated with on-line calibration.
- **Offset Errors:** Each projection path output will also yield a non-zero output for a zero input signal, due to DC shifts and offsets accumulated through the signal path. Transistor mismatch in the integrator, residual charge injection from the multiplier switches and *reset*/*read* integrator switches contribute to this error. Harmonic amplitude outputs then appear with a static shift in value. Amplifier offset calibration along with correlated double-sampling techniques can be effective for reducing this type of static error.
- **Random Errors:** Finally, 1/*f*, thermal and switching noise will add random variation to the input signal. The total effect may be modeled as a random variable added to each output, *y_Ik_* and *y_Qk_*. An alternate model of these sources is to add noise with an equivalent total spectral density to the input signal.

### 4.3. System Classification Rates with Hardware Errors

**Figure 10.**
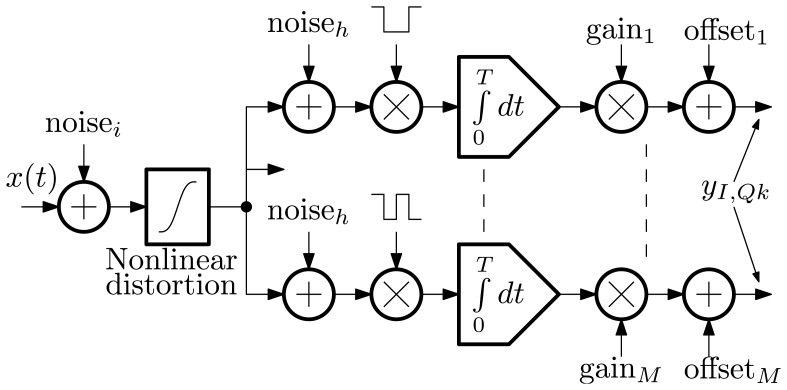
Simulation model of hardware error sources.

Verification of the technique, including estimated analog hardware error sources, was conducted by replacing the explicit computation of Equation (2) with the system described by Figure 10. Noise was added in two locations to separately model the noise contribution of the input buffer amplifier and the equivalent input noise of each multiply-integrate channel. Instances of *noise_h_* have identical spectral densities, but are generated independently.

The low FF of the vehicle classification case study presents very severe hardware requirements (much longer time constant) and correspondingly larger potential errors than those of bearing fault detection in Section 3.2. Hence, for the feasibility study, we will present results for the vehicle classification task. Initialization data for the error modeling was obtained from a system design implemented in a standard 0.13 *μ*m CMOS process [33]. Because the amplifier/integrator distortion can be estimated *a priori*, event windows randomly selected for training the FNN were subjected to the same memoryless nonlinear distortion function, extracted from transistor-level simulation.

Neural network training was performed on a system instance whose *σ*_gain_ and *σ*_offset_ values and noise magnitudes were set to zero—*i.e.*, ideal calculations. Signal-to-noise ratios (SNR) were set by varying the input amplitude and adding noise of density and power extracted from transistor-level simulations. Therefore, the total vehicle recording is considered “signal” for these simulations. System-level vehicle classification rates were then simulated at two noise levels and over a range of gain and offset error levels. For each system instance generated for testing, which models a specific silicon die sample, the gain and offset random variables (RV) were generated from Gaussian distributions of varying standard deviations. The gain RV had a mean of × 1, and the offset RV had a mean of 0 mV.

**Figure 11.**
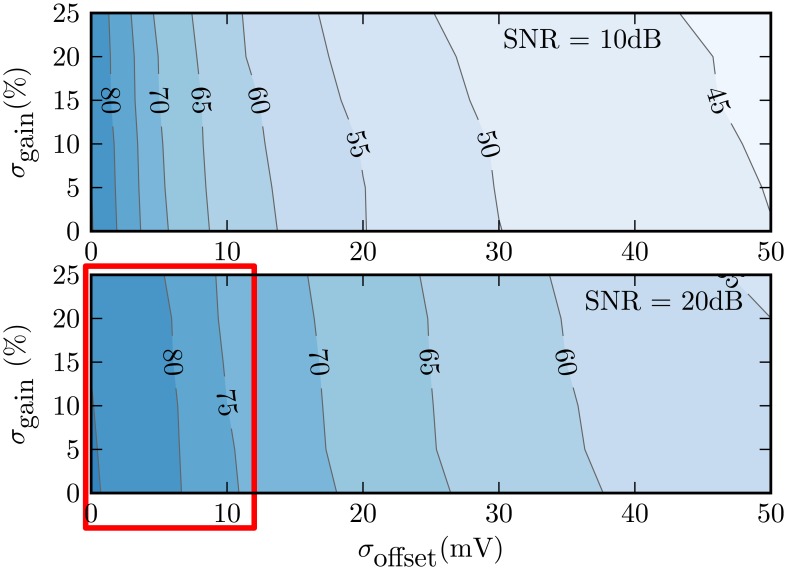
Average classification rate variation over gain/offset standard deviation and added noise values for the vehicle classification task.

Figure 11 plots a contour map of average system-level classification results for a range of gain/offset standard deviations and noise levels for the vehicle classification task. It is clear that gain variations with standard deviations up to 25% have little effect on classification performance. However, classification rates are much more sensitive to offset variations. Offsets have the effect of consistently over-estimating the signal energy at that harmonic, even when there is insignificant signal content at that particular frequency.

Achieving acceptable single-event average classification rates of 75% or above at moderate 20 dB SNR requires offset deviations less than about 10 mV, as indicated by the boxed area in Figure 11. For full-scale circuit outputs of ±1.2 V, this represents relative offset variations on the order of 1%. The boxed region of Figure 11 then indicates the range of errors allowable to maintain good system-level performance. This region, therefore, sets the target hardware design parameters for the vehicle classification application of Section 3.1.

**Figure 12.**
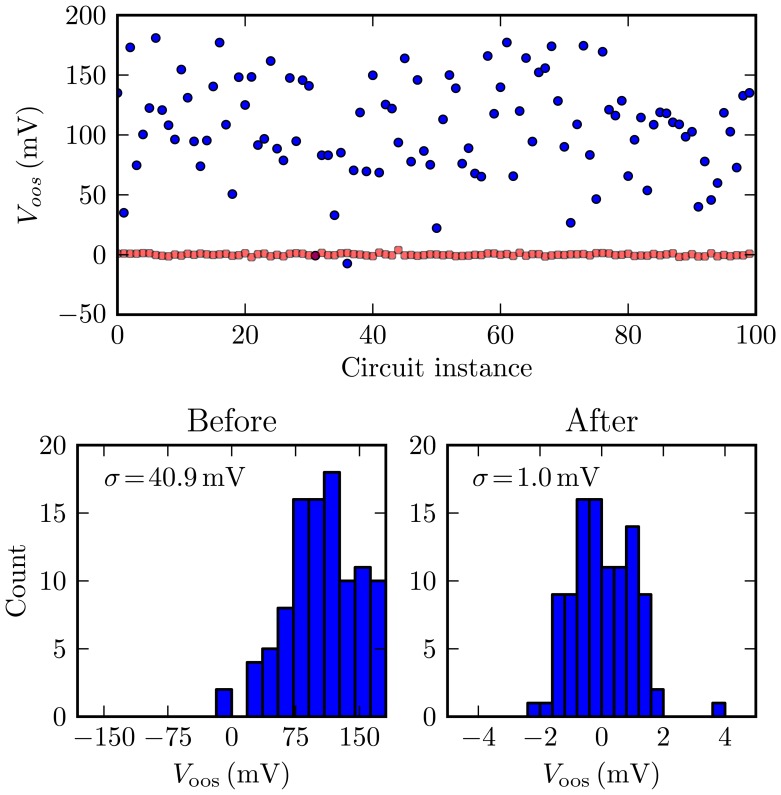
Scatter plot and histogram showing pre- and post-calibration integrator offset error for 100 Monte Carlo simulations. Note the horizontal scale change for the right post-calibration histogram. Adapted from [33].

To determine whether these offset values are feasible, we performed Monte Carlo simulations of a transistor-level integrator design, which included offset calibration circuitry. Figure 12 summarizes the offset error from 100 random instances, both before and after internal calibration [33]. Circuit calibration brought the unacceptable offset standard deviation of 41 mV down to around 1 mV. Because this simulated post-calibration error is well below the estimated 10 mV upper bound, it is expected that most chip instances from this design would be able to achieve acceptable system-level performance [33]. Such on-chip calibration is essential to achieve (offset) errors within the feasible region of Figure 11.

The integrators utilized for this study dissipated 200 nW each, when tuned for a 5 Hz FF [33]. Thus, with two projections per harmonic and the parameters *M* = 50 with a 200 ms integration time of Case Study I, the feature vector computation would consume 4 *μ*J of energy After computation, the system ADC would sample the projection results and pass the data to the classifier or other back-end system processing. This energy consumption is three orders of magnitude less than the 5,100 *μ*J FFT-based feature vector computation energy measured in [17] for a similar military vehicle classification application using commercially available components.

On-chip custom FFT circuitry, such as [34], can naturally use less energy than a software-based computation. The quoted 116 nJ per 128-point transform does not include the system overhead for loading the input data or reading the result from the module. For the 128-point custom hardware, this requires loading 128 values and reading 128 values back into the system processor. Common processors utilized in wireless sensor systems include the MSP430 series [35], which requires approximately 1.5 nJ per instruction to move data in memory. This overhead is greater than the computation energy itself and significantly erodes the net energy advantage of a special module. Unlike an FFT computation, the AHT scheme does not require computing the complete set of coefficients, allowing the unused channels to be powered-down for increased energy savings. Circuitry used in the AHT can also be re-purposed for other signal processing tasks when not actively projecting, reducing the increased die area penalty of special-purpose circuitry. For example, due to their continuous-time operation, the channels can be used as a real-time spectral energy detector to trigger further digital processing—FFT-based techniques by their nature require the system processor to be active.

## 5. Conclusion

In this paper, a transform suited to parallel, low-power analog implementation was presented. The Analog Harmonic Transform allows efficient extraction of only the narrow spectral features needed by the back-end processing without requiring transforming of the entire signal bandwidth at once, like FFT-based approaches. It does, however, provide the data to easily back-resolve the Fouriér coefficients if required by the back-end processing.

The AHT was tested on two monitoring applications using different modalities (acoustic and vibration signals) and a wide range of fundamental frequencies with good discrimination using neural network classifiers. Hardware modeling simulations show that the effect of implementation errors can be small enough with proper design and calibration to allow reliable detection and classification to be feasible with the proposed low-power approach.

## Figures and Tables

**Table 1.** Comparison of digital real-valued operations to compute Fouriér series coefficients from the Analog Harmonic Transform (AHT) back-substitution (*M* = 32, 64) or real-input Fast Fouriér Transform (FFT) algorithm (*N* = 64, 128). FS, Fouriér series; RSR, Real Split-Radix.

**FS Coeff's**	**AHT**		**RSR-FFT**		**SavingsTotal**

	**Mult+Add**	**Total**	**Mult+Add**	**Total**	
1–32	74+74	148	98+420	518	**71%**
3–32	52+52	104	98+420	518	**80%**

1–64	194+194	388	258+1,028	1,286	**70%**
4–64	82+82	164	258+1,028	1,286	**87%**

**Table 2.** Military vehicle single-event detection, false alarm and classification rates, from [23].

**Number of Harmonics, M**			**25**	**50**	**100**
	**Vehicle**	**Type**	$\hat{FF}=5\text{Hz}$		
Detection rate (%)	Leopard 1	TH	92.87	96.20	95.83
	Leopard 2	TH	81.25	90.91	91.85
	Jaguar	TH	80.09	90.16	88.79
	M48	TH	88.09	95.62	95.47
	Wiesel	TL	77.42	82.95	86.62
	Fuchs	WH	81.89	88.79	87.66
	Hermelin	WH	53.04	66.77	64.82
	MB1017	WL	49.39	59.02	65.94
	Unimog	WL	56.81	64.20	63.77

False alarm rate (%)	Leopard 1	TH	1.33	0.89	1.09
	Leopard 2	TH	5.15	2.48	2.69
	Jaguar	TH	3.87	2.30	2.14
	M48	TH	2.08	0.63	0.55
	Wiesel	TL	3.29	2.27	2.21
	Fuchs	WH	2.49	1.98	1.64
	Hermelin	WH	1.58	1.26	0.89
	MB1017	WL	2.10	1.54	1.70
	Unimog	WL	1.04	0.56	0.59

Classification rate (%)			80.00	87.73	88.14

Type key: T = tracked, W = wheeled, H = heavy-weight, L = light-weight.

**Table 3.** Bearing fault single-event detection, false alarm and classification rates, from [23].

**Number of Harmonics, M**		**10**	**20**	**40**
	**Bearing Fault**	$\hat{\text{FF}}=100\text{Hz}$		
Det. (%)	No fault	99.68	99.83	100.00
	Ball fault	83.80	94.34	98.79
	Inner race fault	86.70	96.04	98.93
	Outer race fault	76.73	94.90	98.49

F.A. (%)	No fault	0.10	0.03	0.00
	Ball fault	9.28	2.16	0.45
	Inner race fault	1.22	0.85	0.33
	Outer race fault	6.23	1.66	0.39

Classification rate (%)		87.20	96.42	99.09
